# SARS-CoV-2 Antibody Responses in Pediatric Patients: A Bibliometric Analysis

**DOI:** 10.3390/biomedicines11051455

**Published:** 2023-05-16

**Authors:** Ionela Maniu, George Constantin Maniu, Elisabeta Antonescu, Lavinia Duica, Nicolae Grigore, Maria Totan

**Affiliations:** 1 Mathematics and Informatics Department, Research Center in Informatics and Information Technology, Faculty of Sciences, “Lucian Blaga” University, 5-7 Ion Ratiu Str., 550025 Sibiu, Romania; george.maniu@ulbsibiu.ro; 2 Pediatric Research Team, Clinical Pediatric Hospital, 2-4 Pompeiu Onofreiu Str., 550166 Sibiu, Romania; 3 Faculty of Medicine, Lucian Blaga University of Sibiu, 2A Lucian Blaga Str., 550169 Sibiu, Romania; elisabeta.antonescu@ulbsibiu.ro (E.A.); nicolae.grigore@ulbsibiu.ro (N.G.); maria.totan@ulbsibiu.ro (M.T.); 4 County Clinical Emergency Hospital, 2-4 Corneliu Coposu Str., 550245 Sibiu, Romania; 5 Clinical Laboratory, Clinical Pediatric Hospital, 2-4 Pompeiu Onofreiu Str., 550166 Sibiu, Romania

**Keywords:** antibodies, COVID-19, bibliometric analysis, disease severity, inflammatory markers, VOSviewer, Bibliometrix

## Abstract

The characteristics, dynamics and mechanisms/determinants of the immune response to SARS-CoV-2 infection are not fully understood. We performed a bibliometric review of studies that have assessed SARS-CoV-2 antibody responses in the pediatric population using Web of Science online databases, VOSviewer and Bibliometrix tools. The analysis was conducted on 84 publications, from 310 institutions located in 29 countries and published in 57 journals. The results showed the collaboration of scientists and organizations, international research interactions and summarized the findings on (i) the measured titers of antibodies (total antibody and/or individual antibody classes IgG, IgM, IgA) against different antigens (C-terminal region of N (N CT), full-length N protein (N FL), RBD, RBD Alpha, RBD Beta, RBD Gamma, RBD Delta, spike (S), S1, S2) in the case of different clinical forms of the disease; and (ii) the correlations between SARS-CoV-2 antibodies and cytokines, chemokines, neutrophils, C-reactive protein, ferritin, and the erythrocyte sedimentation rate. The presented study offers insights regarding research directions to be explored in the studied field and may provide a starting point for future research.

## 1. Introduction

The virus that caused the pandemic at the end of 2019 is SARS-CoV-2 (Severe Acute Respiratory Syndrome coronavirus 2). SARS-CoV-2 is a single-stranded RNA virus belonging to the B lineage of the beta-coronavirus family with the ability to encode 14 open reading frames (ORFs). The ORFs have the capacity to encode 4 structural proteins (tip [S], nucleocapsid [N], envelope [E], and membrane [M] proteins), 16 nonstructural proteins, and 9 putative accessory proteins (ORF3a, 3b, 6, 7a, 7b, 8, 9b, 9c and 10) [1].

When the SARS-CoV-2 virus enters the human body, the host’s immune system recognizes it, initiates and induces an immune response (develops proteins called antibodies) in order to attack/eliminate the virus [1,2,3].

The characteristics, dynamics and mechanisms/determinants of the immune response to SARS-CoV-2 infection are not fully understood. Understanding the antibody response to SARS-CoV-2 infection might provide important insights for planning clinical interventions/therapeutics and policy interventions (identifying individuals at risk of adverse outcomes, preventing progression to severe disease, improving treatment, cost-effectiveness/advantages of vaccination). The immune response to SARS-CoV-2 infection is generally determined by the antibodies developed against COVID-19 (specific immunoglobulin M (IgM), A (IgA) and G (IgG)) against different antigens (spike (and its subunits S1, S2), nucleocapsid, receptor-binding domain (RBD)).

There have been a number of research studies (and are continually being updated) that have included analyses of the human immune response to SARS-CoV-2 in adult patients; this is in comparison with the pediatric population, where the evidence is more scarce.

The aim of this study was to perform a bibliometric review of studies that have assessed SARS-CoV-2 antibody responses in the pediatric population in order to provide a comprehensive overview of the state of, and trends observed in, the research conducted in the analyzed field. We identify the main areas of research, the collaboration of scientists and organizations, international research interactions and the immunological features associated with the different clinical forms of SARS-CoV-2 infection and its inflammatory markers.

## 2. Materials and Methods

**Figure 1:**
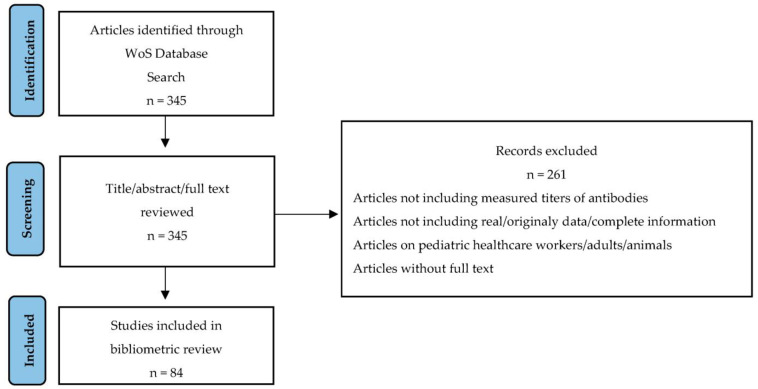
PRISMA flow chart of the study selection process.

For this study, a search of the literature was performed using the Web of Science Core Collection (WoS) online databases in November 2022 in order to identify scientific contributions, including the assessment of SARS-CoV-2 antibody responses in pediatric patients. The search strategy included the keywords (antibod* or IgA or IgG OR IgM) and COVID and (pediatric OR children) and (prevalence or leve* or tite*). The search identified publications that contained the mentioned terms in their title, abstract or keywords. The retrieved studies were than evaluated by the authors to ensure that only articles related to the SARS-CoV-2 antibody responses in children were included in the analysis. Only article-type documents that measured the titres of antibodies against different tested antigens were included. For each included study, the publication title, abstract, keyword/keyword plus, authorship, publication year, journal title, language, journal category and number of total citations were extracted. The process of study selection is described using a PRISMA flow diagram in Figure 1.

The bibliometric analysis and visualizations were performed by using Visualization of Similarities viewer (VOSviewer) software (VanEck andWaltman, Center for Science and Technology Studies of Leiden University [4,5]), a tool that has been used in studies in various areas of research [6,7,8,9,10,11,12,13,14,15,16,17]. Bibliographic techniques, such as citation and co-citation analysis, bibliographic coupling and co-occurrence analysis, were used to create collaboration networks and text mining. In the case of inter-institutional or country collaboration networks, the number of publications (that have authors from different institutes or countries) was considered as a metric for collaboration. The Bibliometrix 3.1 package (Aria and Cuccurullo, University of Naples and University of Campania’s Luigi Vanvitelli, Italy, [18]) and RStudio environment (CRAN, https://cran.r-project.org/ (accessed on 1 November 2022)) were used to create the historiographical citation network, the co-citations network statistics, and the top authors’ production over time plots.

## 3. Results

**Figure 2:**
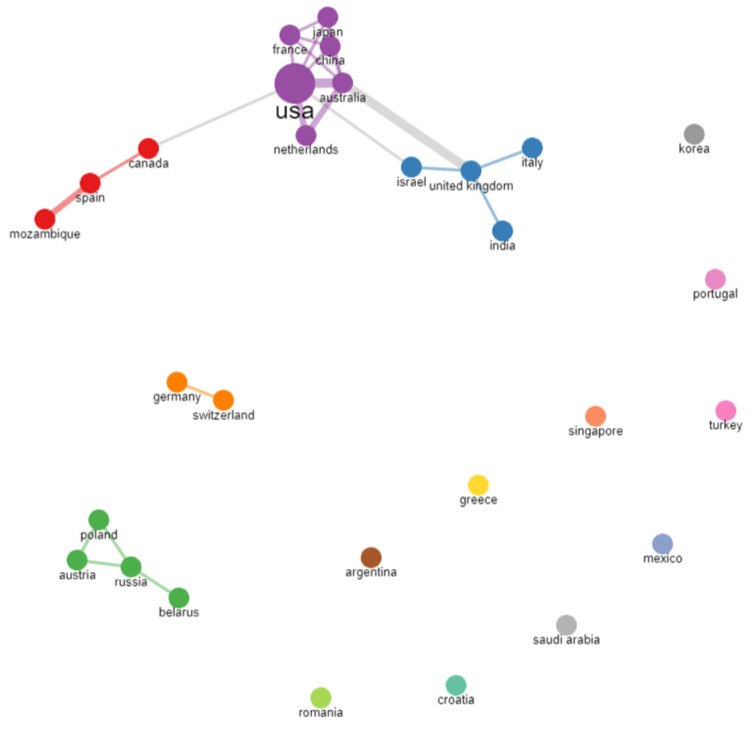
Bibliometrix collaboration network between countries.

Of the 345 studies retrieved from the search, 84 were included in the bibliometric analysis. A total of 29 countries had published documents in the research field. Among the most productive countries were North American countries (USA, Canada), Asian countries (China, Israel), European countries (Italy, UK, Poland, Spain, France, Germany, Netherlands, Turkey, and Russia), one Oceania country (Australia), and one South American country (Argentina). The scientific collaboration between countries is presented in Figure 2. The links between the countries represent co-authorships and the widths of the links represent the different frequencies of collaboration (thicker links reflect more collaboration between the two countries). As indicated in the graph, clusters of interconnected countries (marked with different colors—purple, red, blue, green, orange), but also isolated/unconnected countries (those that did not have any international collaboration, although some of them, such as Turkey, had four publications), can be observed.

**Figure 3:**
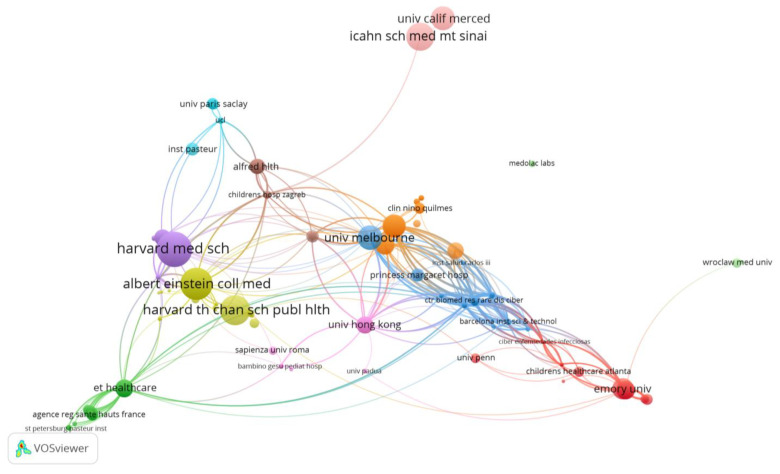
VOSviewer network visualization map of institutions/organizations (type of analysis: citations, weights—citations, largest set of connected items—236).

A total number of 310 organizations were found to have published papers related to the topic of SARS-CoV-2 antibody responses in pediatric patients. The most productive and cited organizations are presented in Table 1 and Figure 3. 

The University of Melbourne (Australia) was found to have published 5 related papers, with 128 citations. The main partners of the organization are the Royal Children’s Hospital and Murdoch Childrens Res. Inst., and other organizations also from Australia, but also organizations from the USA (Univ. of Michigan, Washington Univ. School of Medicine in St. Louis, Icahn School of Medicine at Mount Sinai), UK (London Sch Hyg and Trop med), China (Univ. Hong Kong, Chinese Univ. of Hong Kong), Netherlands (University of Amsterdam), France (Univ. de Versailles Saint-Quentin, Univ. Paris Descartes), and Japan (Ishikawa Prefectural Univ.). The researches focused on a comparative analysis between the coronavirus antibody responses present in children and adults/elderly people with COVID-19 and/or healthy patients. 

Emory University (Atlanta, Georgia, USA) was also found to have published 5 related papers with 93 citations. The main partner of the organization is the Children’s Healthcare Atlanta hospital and other organizations also from Atlanta or from other USA states (Missouri, Arizona, Florida, Tennessee, Virginia, Georgia, Texas); the researches mainly concentrated on inflammatory markers and the SARS-CoV-2 antibody profiles of children with multisystem inflammatory syndrome (MIS-C) (and/or comparison with patients with acute/symptomatic COVID-19, healthy controls, Kawasaki disease, and also with a mouse model).

Next in the hierarchy of the top productive organizations were two universities from China: the University of Hong Kong and the Chinese University of Hong Kong, with 5/4 related publications, of which 4 are joint articles (62/61 citations). The main partners of the organization are the Hospital Authority of Hong Kong, the Queen Mary/Princess Margaret/Prince of Wales/Queen Elizabeth hospitals and other organizations also from China or from the USA and Australia. The researchers investigated humoral and cellular (T cell) responses and the long-term persistence of SARS-CoV-2-neutralizing antibody responses in recovered children and adolescents.

The collaboration between several American institutions, Harvard Medical School, Massachusetts General Hospital and Brigham and Women’s Hospital, led to the publication of two of the top ten most cited articles related to the analyzed topic [19,20] and implicitly to the positioning of these institutions in the top of the most cited organizations. In addition, the next four (also American) institutions in this hierarchy were as follows: the Albert Einstein College of Medicine, the Children’s Hospital at Montefiore, Montefiore Medical Center and Yale University. These institutions collaborated in the creation of another two of the top ten most cited articles [21,22]. 

**Figure 4:**
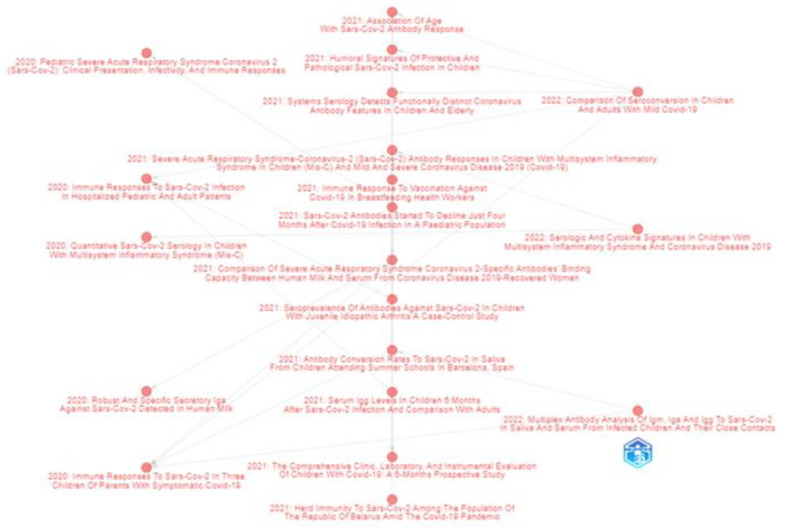
Bibliometrix historical analysis of direct citation of top-cited papers related to the researched topic. The nodes in the figure represent documents and the directional arrow represents the citation association between two documents.

**Figure 5:**
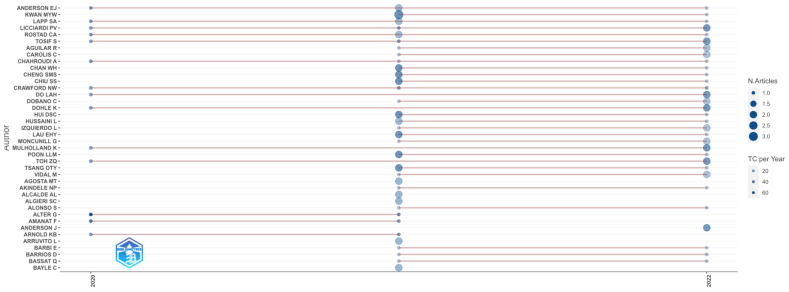
Top authors’ production over time in the research field. The size of the circle is directly proportional to the number of documents, the shade of the color represents the number of citations; TC per Year—Total Citation per year.

The top 20 highly cited articles are presented in Table 2. The historiographic analysis presented in Figure 4 shows the genealogic structure mapping of the cited papers (bibliographic antecedents and descendants). The relevant authors, based on their publication number, are presented in Figure 5. 

In total, 57 journals published research articles in the field. The journals with the highest number of publications were Nature Communications (4 articles), Frontiers in Pediatrics (3), JAMA Network Open (3), Pathogens (3), Frontiers in Immunology (3), JCI Insight (3), eBioMedicine (3) and the Journal of Clinical Medicine (3); meanwhile, the journals that had the highest number of citations were the Journal of Pediatrics (1 article/192 citations), Science Translational Medicine (1/167), Nature Communications (4/140) and iScience (1/119).

**Figure 6:**
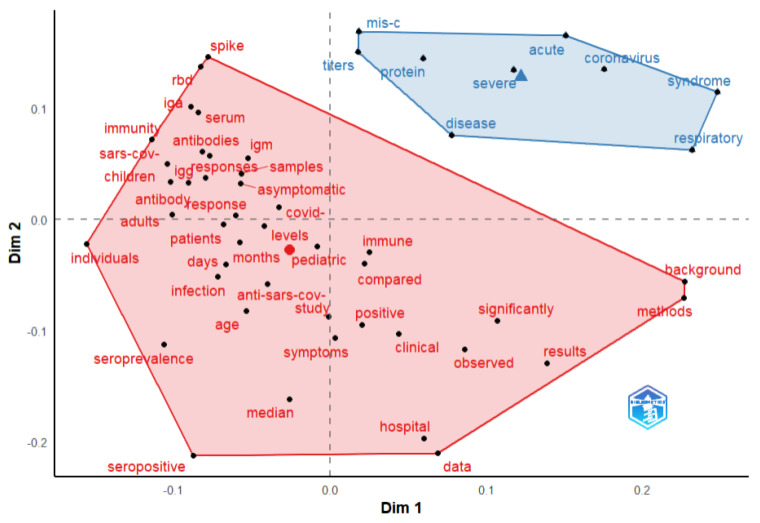
Bibliometrix conceptual structure map of terms from documents abstract. Factorial analysis method—multidimensional scaling.

Factorial analysis was used to identify the topics in the publications. Based on a keywords analysis, two clusters were identified (Figure 6). The red cluster includes the main terms related to the antibody responses and clinical characteristics exhibited in COVID-19 symptomatic and asymptomatic pediatric patients (included terms: IgG, IgM, IgA, spike, RBD, seropositive, seroprevalence, immunity, clinical, symptoms), while the blue cluster refers to SARS-CoV-2 antibody responses in the case of severe and MISC pediatric patients.

## 4. Discussion

There have been a large number of articles published on COVID-19 since the beginning of the pandemic. Our bibliometric review focused on a specific portion of the scientific literature: those publications on the topic of SARS-CoV-2 antibody responses in the pediatric population. The analysis carried out using VOSviewer and Bibliometrix showed publication patterns, as well as country/organization/author collaborations. The country networks showed that the USA, Republic of China, and Italy were the most prolific countries in the field, while the most cited ones were the USA, Canada, and Australia. Moreover, collaboration patterns can be observed between the USA–Netherlands–Australia–Japan–France–China; UK–Italy–India–Israel; and Russia–Austria–Poland–Belarus. In Australia, the University of Melbourne, in the USA, Emory University, and in China, the University of Hong Kong and the Chinese University of Hong Kong, collaborated on research with other institutions; meanwhile, collaboration between the Harvard Medical School–Massachusetts General Hospital–Brigham and Women’s Hospital, and the Albert Einstein College of Medicine–Children’s Hospital at Montefiore–Montefiore Medical Center–Yale University, led to the publication of articles on the list of top ten most cited articles related to the analyzed topic. An analysis of the studies conducted by research centers/institutes revealed a focus on performing comparative analyses between the coronavirus antibody responses present in children and adults/elderly with COVID-19, and/or healthy patients (University of Melbourne, Australia), the inflammatory markers and SARS-CoV-2 antibody profiles of MIS-C patients (University of Melbourne, USA), humoral and cellular (T cell) responses, and the long-term persistence of SARS-CoV-2-neutralizing antibody responses in recovered children and adolescents (University of Hong Kong and the Chinese University of Hong Kong, China). Articles were published in journals concerning pediatrics, medicine in general and internal, research and experimental science, biochemistry and molecular biology, cell biology, microbiology, immunology and multidisciplinary sciences. The results of the keywords analysis identified two clusters, including terms related to the antibody responses and clinical characteristics exhibited in COVID-19 symptomatic and asymptomatic pediatric patients, and terms related to SARS-CoV-2 antibody responses in the case of severe and MISC pediatric patients.

### 4.1. Antibodies Levels and Disease Severity

In the analyzed studies, the SARS-CoV-2 antibodies were detected in serum/plasma, mucosa/nasal fluid, saliva, breast milk, and cerebrospinal fluid.

In the study of [40], the positive rates of anti-spike IgG, antiSARS-CoV-2 IgG, and of neutralizing antibodies in the case of symptomatic children gradually increased and reached 100% within 14–28 days after onset, and there was a plateau at two months after the onset of COVID-19 symptoms. In contrast, positive anti-S IgA were detected within 14–28 days after onset in both symptomatic (75%) and asymptomatic children (60%).

Disease severity is associated with a greater antibody response [37,41,42,43]. Akindele et al. [43] reported elevated levels of IgG titers in MISC patients (median 6.75, IQR 0.84–10.88) compared with acute COVID-19 patients (median 2.98, IQR 0.28–5.76), but the difference was not statistically significant (*p* = 0.241). A study from India [44] reported a median IgG antibody titer of 54.8 AU/mL (range 11.09–170.9), with significantly higher levels among children with MISC (median 60.3 AU/mL, range: 12.3–170.9 vs. children without MISC—median 54.8 AU/mL, range 11.0–144.3), and with significantly lower levels among children with MISC needing intensive care (PICU) (median 45.72, range: 18.92–156.37 vs. children who did not require PICU—median 81.28, range: 12.32–170.21). The study [45] reported high anti-spike IgG and IgA levels (in the sera and saliva of young infants compared with their parents) and low but detectable SARS-CoV-2-specific CD4^+^ and CD8^+^ T cell responses in young infants. In terms of IgM, the study from India [44] reported positive SARS-CoV-2 IgM antibodies in only 14% of children from the study. All children with positive IgM had no features suggestive of MISC and half of them also had a positive IgG test result. The median IgM antibody titer was 31.1 AU/mL (range 11.9–139.9). The study [24] reported that 100% of MIS-C and 80% of COVID-19 patients had detectable IgM antibodies against SARS-CoV-2 RBD (indicating a recent SARS-CoV-2 infection.)

The results of another study [36] showed that the IgM and IgG antibody levels were significantly lower at 180 days after infection in children (IgM median 0.74 (0.64–1.01) AU/mL, IgG median 16.53 (9.1–24.1) AU/mL) compared with levels at 30 days (IgM median 1.29 (1.02–1.47) AU/mL, IgG median 90.61 (71.5–101) AU/mL). Moreover, at 180 days after infection in children, these were at a lower level compared to their parents (parents IgM median 0.83 (0.53–1.19) AU/mL, IgG median 92.7 (44.1–163.3) AU/mL).

In study [22], both anti-SARS-CoV-2 IgA and IgG were quantified in nasal fluid and showed similar levels. In the study of [46], breast milk and serum anti-SARS-CoV-2 IgG and IgA, in the case of vaccinated breastfeeding women, were reported. The breast milk IgG and IgA levels were highly correlated (r = 0.89; r = 0.83, *p* < 0.001) to serum IgG and IgA levels.

Higher antibody levels were observed in symptomatic children in comparison with asymptomatic cases. In study [47], symptomatic children had significantly higher IgM levels (N CT, N FL, RBD, RBD Alpha, RBD Beta, RBD Gamma, RBD Delta, S, S1, S2) and IgG levels (N FL, RBD, RBD Alpha, RBD Beta, RBD Delta, RBD Gamma, S, and S2) in comparison with asymptomatic cases. 

Children with MISC had higher antibody levels than non-MISC cases. In study [47], pediatric patients with MISC had significantly higher IgG levels (N FL, N CT, the S, S1, S2, RBD located in S1, from the Wuhan strain, RBD Alpha, RBD Beta, RBD Gamma, RBD Delta), higher IgA levels (for all tested antigens, except N-CT), and higher IgM levels (RBD, RBD Alpha, RBD Delta, S1) in comparison with non-MISC cases. In study [19], elevated IgM and IgG SARS-CoV-2 levels were observed in severe MISC cases in comparison with mild MISC cases. In a prospective study [24] analyzing children with MISC, symptomatic COVID-19, Kawasaki disease (KD), and hospitalized pediatric controls, higher levels of IgM, RBD IgG, full-length spike, and nucleocapsid protein antibody titers were encountered in the case of MISC in comparison to other groups. In addition, study [29] reported elevated levels of IgG antibody titers against SRBD and full-length S, and IgA antibody titers against full-length S (but not S-RBD) in patients with MISC compared to patients with severe COVID-19 (majority of whom had undetectable levels of the spike or nucleocapsid proteins of IgG antibodies). In the case of IgM antibody titers and anti-SARS-CoV-2 N antibodies, the differences were not statistically significant. The same study also reported no differences between the IgG antibody (directed to spike or nucleocapsid proteins) levels in children with or without immunodeficiency.

A correlation between saliva and plasma antibodies has been detected in COVID-19 pediatric patients [48]. The collection of SARS-CoV-2-specific saliva antibodies is a cost-effective, non-invasive and easy assay that may be used to determine levels of immunity after infection or immunization with COVID-19 vaccines, at an individual or population level [48,49].

Immunoglobulin A has an important role in fighting infectious pathogens from the point of entry (respiratory and digestive system), acting as an immune barrier; it has the ability to neutralize them before they enter the body and bind to epithelial cells [50]. A systematic review and meta-analysis of the role of IgA in COVID-19 diagnosis or severity, including 38 scientific articles from PubMed database, observed that IgA production correlates with disease severity (IgA is produced more effectively in patients after severe disease compared with mild or asymptomatic patients), and concluded that further studies should establish the roles of mucosal/systemic IgA responses in the protection/immunopathology of COVID-19 [51]. Mucosal vaccination therapy may be an effective treatment strategy by which to induce a local protective immunity within the mucosa. 

### 4.2. Relationship between SARS-CoV-2 Antibodies and Inflammatory Markers

Several studies have analyzed the correlations between SARS-CoV-2 antibodies and cytokines, chemokines, neutrophils, C-reactive protein (CRP), ferritin (a marker of macrophage activation) and the erythrocyte sedimentation rate (ESR—systemic inflammation marker). 

Some studies have analyzed antibody and cytokine responses in COVID-19 pediatric patients in order to investigate the relationship between the early responses of inflammatory cytokines, the late-stage responses of anti-SARS-CoV-2 IgG/IgM antibodies, and disease severity. The invasion of SARS-CoV-2 leads to the activation of innate immunity, and to determinate host cells initiating the inflammatory response and the release of large amount of cytokines and chemokines [52,53,54,55]. The results of a study analyzing anti–SARS-CoV-2 IgG/IgA, cytokines, and total protein using nasal mucosal secretions [22] indicated that there was not a strong correlation between antibodies and cytokines (IL-1, IFN-α2, IFN-γ, IP-10, IL-8, IL-1α, IL-1β, IL1-RA, MCP1), except for IL-18. There was a strong inverse correlation between IL-18 and anti–SARS-CoV-2 IgG (anti-S1, anti-S2, anti-RBD and anti-NC) and IgA (anti-S1, anti-S2, anti-RBD, anti-NC). In a study [56] analyzing SARS-CoV-2 antibody profiles in a case series of five children with neuropsychiatric symptoms associated with COVID-19, the antibodies of four SARS-CoV-2 antigens (S1 N-terminal domain (NTD), the S1 receptor binding domain (RBD), full-length spike (S), nucleocapsid (N)) correlated with pro-inflammatory cytokines and chemokines (in this study, the SARS-CoV-2 antibodies, cytokines, and chemokines were collected from cerebrospinal fluid). In particular, the antibodies to the N protein correlated most strongly with pro-inflammatory cytokines (GM-CSF, IL-2, IL-8, IL-13, IP-10, MCP-1, MIP-1 β, and TNF-α). Another study [43] reported higher levels of 14 of 37 cytokines/chemokines (IL-1RA, IL-2RA, IL-6, IL-8, tumor necrosis factor-α, IL-10, IL-15, IL-18, MCP-1, IP-10, MIP-1α, MCP-2, MIP-1β, eotaxin, regardless of age or sex, duration of symptoms, length of hospital stay, nasopharyngeal viral RNA levels) and high IgG titers in children with MIS-C compared to those with acute COVID-19. The study of [37] suggests a differentiation (that might be used at hospital admission) between symptomatic (mild/moderate) and asymptomatic patients based on the neutrophil expression of CD64 and serum levels of IgG antibodies (spike protein of SARS-CoV-2); there were higher levels in symptomatic patients compared with asymptomatic patients. A study from India [44] also reported increased neutrophil counts and higher levels of IgG antibodies in children with MISC compared with COVID-19 patients without MISC. Pierce and colaborators [22] reported that SARS-CoV-2-specific IgA and IgG concentrations were detected in nasopharyngeal samples, being negatively correlated with mucosal IL-18 levels. IL-18, a cytokine predominantly produced by macrophages, under the action of the NLRP3 inflammasome is cleaved to its active form and then the production of IFN-γ takes place. The early release of IL-18 is thought to moderate the adaptive response, with elevated serum levels of IL-18 being associated with disease severity. IFN-β1 was used for the early treatment of COVID-19 according to the study [57]. The results showed an association between disease severity and the joint action of various cytokines and chemokines. These findings of cytokine/chemokine dysregulation are consistent with results from studies on adults [55,58,59,60,61].

In study [19], in the case of MIS-C patients, NT-proBNP significantly positively correlated with IgG SARS-CoV-2 RBD (*p* = 0.008), but not with IgM SARS-CoV-2 BRD (*p* = 0.73). Correlation between SARS-CoV-2 IgG and NT-proBNP could indicate mechanism/disease severity. In the same study, no correlation between CRP and IgM/IgG SARS-CoV-2 RBD was encountered, while ferritin positively correlated with IgM/IgG SARS-CoV-2 BRD (*p* = 0.03/0.10). Ferritin (high) levels and their correlation with SARS-CoV-2 serology suggest an interplay between SARS-CoV-2 antibodies and monocyte/macrophage activation in MIS-C patients. In study [24], also in the case of MIS-C patients, RBD IgG antibody levels correlated with the erythrocyte sedimentation rate (*p* = 0.046), but not with CRP.

Overall, this study provides insights into the research directions in the field of SARS-CoV-2 antibody responses in the pediatric population and may provide a starting point for the future research directions of practitioners/policymakers/researchers/patients/organizations, etc. The study was limited to documents published in English, from WOS, up to November 2022. Searches using other databases, such as Scopus, PubMed, EMBASE, Google Scholar, Dimension, etc., and using a different time range (taking into account the growing body of scientific literature) may give a different set of records.

## Figures and Tables

**Table 1:** Top productive and cited organizations.

Rank	Organizations Publications	Rank	Organizations Citations	Rank	Organizations Affiliations
1	Univ Melbourne (5)	1	Harvard Med Sch (257)	1	Emory Univ (18)
1	Emory Univ (5)	2	Brigham and Women’s Hospital (252)	2	Univ Hong Kong (17)
1	Univ Hong Kong (5)	2	Massachusetts Gen Hosp (252)	3	Johns Hopkins Univ (16)
2	Chinese Univ Hong Kong (4)	3	Albert Einstein Coll Med (214)	4	Inst Pasteur (14)
2	Childrens Healthcare Atlanta (4)	3	Child. Hosp Montefiore (214)	5	Univ Bristol (11)
3	Harvard Med Sch (3)	3	Montefiore Med Ctr (214)	5	Univ Melbourne (11)
3	Univ Amsterdam (3)	3	Yale Univ (214)	5	Univ Penn (11)
3	Murdoch Child. Res Inst (3)	4	Harvard Th Chan Sch Publ Hlth (192)	6	Univ Barcelona (10)
3	Royal Child. Hosp (3)	5	Hosp Sick Child. (167)	7	Univ Med Ctr Hamburg Eppendorf (8)
3	Hosp Author Hong Kong (3)	6	Icahn Sch Med Mt Sinai (163)	8	Hosp Author Hong Kong (7)
3	London Sch Hyg and Trop med (3)	7	Univ Melbourne (128)	8	Hosp Infantil Mexico Dr Federico Gomez (7)
3	Univ Bristol (3)	8	Univ Calif Merced (119)	8	Med Univ Lublin (7)
3	Barcelona Inst Sci and Techno (3)	9	Univ Amsterdam (113)	8	Royal Child. Hosp (7)
3	Huazhong Univ Sci and Techno (3)	10	Murdoch Child. Res Inst (109)		
3	Queen Mary Hosp (3)				

**Table 2:** Top 20 highly cited articles.

First Author Year [Ref.]	Document Title	Journal	Citations
Yonker 2020 [19]	Pediatric Severe Acute Respiratory Syndrome Coronavirus 2 (SARS-CoV-2): Clinical Presentation, Infectivity, and Immune Responses	JOURNAL OF PEDIATRICS	192
Pierce 2020 [21]	Immune responses to SARS-CoV-2 infection in hospitalized pediatric and adult patients	SCIENCE TRANSLATIONAL MEDICINE	167
Fox 2020 [23]	Robust and Specific Secretory IgA Against SARS-CoV-2 Detected in Human Milk	ISCIENCE	119
Rostad 2020 [24]	Quantitative SARS-CoV-2 Serology in Children With Multisystem Inflammatory Syndrome (MIS-C)	PEDIATRICS	71
Yang 2021 [25]	Association of Age With SARS-CoV-2 Antibody Response	JAMA NETWORK OPEN	68
Bartsch 2021 [20]	Humoral signatures of protective and pathological SARS-CoV-2 infection in children	NATURE MEDICINE	60
Tosif 2020 [26]	Immune responses to SARS-CoV-2 in three children of parents with symptomatic COVID-19	NATURE COMMUNICATIONS	56
Pierce 2021 [22]	Natural mucosal barriers and COVID-19 in children	JCI INSIGHT	47
Selva 2021 [27]	Systems serology detects functionally distinct coronavirus antibody features in children and elderly	NATURE COMMUNICATIONS	44
Cohen 2021 [28]	SARS-CoV-2 specific T cell responses are lower in children and increase with age and time after infection	NATURE COMMUNICATIONS	37
Anderson 2021 [29]	Severe Acute Respiratory Syndrome-Coronavirus-2 (SARS-CoV-2) Antibody Responses in Children With Multisystem Inflammatory Syndrome in Children (MIS-C) and Mild and Severe Coronavirus Disease 2019 (COVID-19)	JOURNAL OF THE PEDIATRIC INFECTIOUS DISEASES SOCIETY	21
Isoldi 2021 [30]	The comprehensive clinic, laboratory, and instrumental evaluation of children with COVID-19: A 6-months prospective study	JOURNAL OF MEDICAL VIROLOGY	19
Lau 2021 [31]	Long-term persistence of SARS-CoV-2 neutralizing antibody responses after infection and estimates of the duration of protection	ECLINICALMEDICINE	19
Woudenberg 2021 [32]	Humoral immunity to SARS-CoV-2 and seasonal coronaviruses in children and adults in north-eastern France	EBIOMEDICINE	18
Shrwani 2021 [33]	Detection of Serum Cross-Reactive Antibodies and Memory Response to SARS-CoV-2 in Prepandemic and Post-COVID-19 Convalescent Samples	JOURNAL OF INFECTIOUS DISEASES	17
Sermet-Gaudelus 2021 [34]	Prior infection by seasonal coronaviruses, as assessed by serology, does not prevent SARS-CoV-2 infection and disease in children, France, April to June 2020	EUROSURVEILLANCE	17
Toh 2022 [35]	Comparison of Seroconversion in Children and Adults With Mild COVID-19	JAMA NETWORK OPEN	16
Bloise 2021 [36]	Serum IgG levels in children 6 months after SARS-CoV-2 infection and comparison with adults	EUROPEAN JOURNAL OF PEDIATRICS	14
Seery 2021 [37]	Blood neutrophils from children with COVID-19 exhibit both inflammatory and anti-inflammatory markers	EBIOMEDICINE	13
Keuning 2021 [38]	Saliva SARS-CoV-2 Antibody Prevalence in Children	MICROBIOLOGY SPECTRUM	13
Vilibic-Cavlek 2021 [39]	SARS-CoV-2 Seroprevalence and Neutralizing Antibody Response after the First and Second COVID-19 Pandemic Wave in Croatia	PATHOGENS	12

## Data Availability

The data are available on request from the correspondent author.
